# Applying Linear and Non-Linear Methods for Parallel Prediction of Volume of Distribution and Fraction of Unbound Drug

**DOI:** 10.1371/journal.pone.0074758

**Published:** 2013-10-07

**Authors:** Eva M. del Amo, Leo Ghemtio, Henri Xhaard, Marjo Yliperttula, Arto Urtti, Heidi Kidron

**Affiliations:** 1 Centre for Drug Research, Faculty of Pharmacy, University of Helsinki, Helsinki, Finland; 2 Division of Biopharmaceutics and Pharmacokinetics, Faculty of Pharmacy, University of Helsinki, Helsinki, Finland; University of Edinburgh, United Kingdom

## Abstract

Volume of distribution and fraction unbound are two key parameters in pharmacokinetics. The fraction unbound describes the portion of free drug in plasma that may extravasate, while volume of distribution describes the tissue access and binding of a drug. Reliable *in silico* predictions of these pharmacokinetic parameters would benefit the early stages of drug discovery, as experimental measuring is not feasible for screening purposes. We have applied linear and nonlinear multivariate approaches to predict these parameters: linear partial least square regression and non-linear recursive partitioning classification. The volume of distribution and fraction of unbound drug in plasma are predicted in parallel within the model, since the two are expected to be affected by similar physicochemical drug properties. Predictive models for both parameters were built and the performance of the linear models compared to models included in the commercial software *Volsurf+*. Our models performed better in predicting the unbound fraction (Q^2^ 0.54 for test set compared to 0.38 with *Volsurf+* model), but prediction accuracy of the volume of distribution was comparable to the *Volsurf+* model (Q^2^ of 0.70 for test set compared to 0.71 with *Volsurf+* model). The nonlinear classification models were able to identify compounds with a high or low volume of distribution (sensitivity 0.81 and 0.71, respectively, for test set), while classification of fraction unbound was less successful. The interrelationship between the volume of distribution and fraction unbound is investigated and described in terms of physicochemical descriptors. Lipophilicity and solubility descriptors were found to have a high influence on both volume of distribution and fraction unbound, but with an inverse relationship.

## Introduction

The extent of drug distribution determines the access of a drug to its sites of action and to other tissues, which might give rise to adverse effects. A primary parameter for drug distribution is the volume of distribution (V_d_) that is defined as.
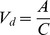
where A is the amount of drug in the body, and C is the drug concentration in plasma (both free drug and protein-bound drug). Volume of distribution is an apparent volume that increases with elevated drug binding in the extravascular space of the body and not an anatomically defined volume. Consequently, extensive drug binding outside the blood vessels leads to increasing values of A/C ratio. As tissue binding of drugs varies considerably, volume of distribution displays a wide range of values. For example, erythropoietin is confined to the vascular space presenting a V_d_ of 4 L (approximately the anatomical volume of vascular space) [1], while hydroxychloroquine with a V_d_ of 49 000 L strongly accumulates into the cells and tissues [2]. Volume of distribution at steady state (V_ss_) is measured at equilibrium, therefore, it describes the molecular tissue binding more reliably than other volume of distribution parameters that are dependent on the time after measurement. V_ss_ depends on the access of the drug to the cells and tissues, its affinity to plasma proteins and tissue components, and number of binding sites in plasma and tissues.

Drug concentration in plasma (C) includes both unbound (C_u_) and protein-bound drug in plasma. However, only the fraction of free drug in plasma permeates across the cellular membranes and vascular walls in most tissues. The free fraction of drug in plasma (f_u_) is described by the ratio C_u_/C. Likewise the drug in the tissues also includes both free (C_uT_) or tissue bound parts. The unbound fraction of drug in tissues is: f_uT_  =  C_uT_/C_T_, where C_T_ is the total drug concentration in the tissue. Drug binding to plasma proteins and tissue components influences drug partitioning between the tissues and plasma. Thus, V_ss_ can be presented using the following equation:
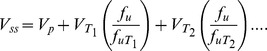
where V_p_ is the anatomical volume of plasma and V_T_ is the true anatomical volume of each tissue. V_ss_ depends on the anatomical volumes of the tissues, and the relative extent of drug binding in the plasma and tissues described as f_u_/f_uT_ ratios.

As volume of distribution describes the extent of drug distribution, it is important to predict its value early in drug development before experimental measuring in humans. V_ss_ in humans may be extrapolated from the *in vivo* animal data that is obtained during the drug discovery process, but computational approaches are useful at early stages before animal data has been collected. The volume of distribution used for computational modeling should be collected from intravenous and not from oral pharmacokinetics studies as in some cases [3], [4]. The benefit of intravenous administration is the defined quantity of the drug that is subject to distribution, which avoids the uncertainty associated with incomplete bioavailability after extravascular administration.

Even though quantitative structure-property relationship (QSPR) has been widely used for prediction of V_ss_ [3]–[16], it remains a challenging problem that has not been adequately solved. The early attempts for predicting volume of distribution were based on small data sets and did not specify the type of volume of distribution that was used as the endpoint or in some cases used several types of volume of distribution for the model building [3]-[8], [11], [13], [14], [17]. In 2008, a major advance was the publication of a clean, manually curated dataset of V_ss_ [18] that subsequently has been used successfully to build predictive models for V_ss_ [12], [16].

The main difference in the work presented here compared to the previously published models of V_ss_ is that we have included another pharmacokinetic parameter, f_u_, to the modeled responses. The f_u_ in plasma depends on the binding affinity and capacity of plasma proteins, which also affect the volume of distribution. The fraction of unbound drug in plasma can be estimated relatively easily *in vitro*, but computational models for predicting f_u_ are also available [19]-[21]. The *VolSurf+* software includes prediction tools for both volume of distribution and plasma protein binding, however, there is limited information of the methodology behind the models and their prediction capacity have not been evaluated in an unbiased manner in the literature. The two parameters, V_ss_ and f_u_, are expected to be affected by similar physicochemical drug properties and our hypothesis was that modeling them in parallel would benefit their prediction. We have applied both linear and nonlinear multivariate approaches: linear partial least square (PLS) regression combined with principal component analysis (PCA) and non-linear recursive partitioning (RP) classification. RP has been shown to perform well when dealing with complex endpoints associated with multiple mechanisms, while PLS allows many responses (in our case V_ss_ and f_u_) to be incorporated in one regression model, but to our knowledge, this approach has not been used previously in pharmacokinetic QSPR modeling.

## Materials and Methods

### 1. Data Set

The initial dataset collated by Obach and co-workers [18] contains 670 compounds with V_ss_ and f_u_ values determined after intravenous administration to healthy people. The collection steps, the quality and the diversity of the data have been meticulously detailed in the publication.

The 2D structures of the compounds were obtained from the ACD/Dictionary version 11 [22] or the PubMed compound database (http://www.ncbi.nlm.nih.gov/pccompound Accessed 2010 October). If the compounds were represented as salts in the 2D structure, the counter ion was discarded. The 3D structures were generated using Concord within *SYBYL 8.0* [23]. A set of 648 drugs with both 2D and 3D structures were obtained. For the remaining 22 compounds in Obach's data set either a 2D structure or minimized 3D structure was not obtained or it was not possible to calculate descriptors from the structures. The V_ss_ of artesunate was corrected to 1.5 L/kg based on the work of White [24]. Furthermore, we excluded ibadronic, pamodronic, risedronic and zoledronic bisphosphonates from the set, since these compounds are sequestered to the bones, preventing their detection in the plasma, and leading to underestimated values of V_ss_ [25]. The antimalarial drugs hydroxychloroquine and chloroquine have V_ss_ values of 700 L/kg and 140 L/kg, respectively. These values are far beyond the range of other V_ss_ values (0.035–60 L/kg) and they were excluded to avoid biasing the model.

**Figure 1:**
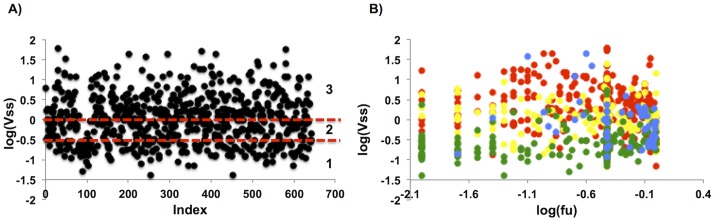
Distribution of compounds in the data set. (A) The distribution of logV_ss_ values in the final dataset. Lines have been draw at 0.3 L/kg and 1 L/kg to indicate the boundaries between the three classes used in the RP models. B) Distribution of compounds based on both log V_ss_ and log f_u_ values and coloring by compound charge (basic-red, neutral-yellow, acidic-green, zwitterionic-blue).

The final data set of 642 drugs (Figure 1) displays V_ss_ values of 0.035–60 L/kg and f_u_ values (541 drugs) of 0.0002–1.

### 2. Calculation of Molecular Descriptors

In this study, molecular descriptors were calculated using *ACDlabs* [26], *Volsurf+* [17] and *MOE* [27]. Input molecular structures were two-dimensional for *ACDlabs* and three-dimensional for *Volsurf+* and *MOE*, for the later Gasteiger-Huckel charges were added. Identical descriptors (i.e. molecular weight, molecular volume) were excluded before combining descriptor sets for modeling. The descriptors that were used for model building are listed in Table 1 and the calculated descriptor values for the data set are available in File S1.

**Table 1:** The descriptors included in modeling.

*ACDlabs* descriptors	*Volsurf+* descriptors						*MOE* descriptors
ALogD5	V	WO1	CW5	POL	%FU4	LgS6	logS
ALogD5.5	S	WO2	CW6	MW	%FU5	LgS6	
ALogD7	R	WO3	CW7	FLEX	%FU6	LgS7	
ALogD7.4	G	WO4	CW8	FLEX_RB	%FU7	LgS7.5	
APSA	W1	WO5	ID1	NCC	%FU8	LgS8	
HDonors	W2	WO6	ID2	DIFF	%FU9	LgS9	
HAcceptors	W3	WN1	ID3	LOGP n-Oct	%FU10	LgS10	
FRB	W4	WN2	ID4	LOGP c-Hex	DRDRDR	LgS11	
Rule Of 5	W5	WN3	CD1	PSA	DRDRAC	L0LgS	
Molar Volume	W6	WN4	CD2	HAS	DRDRDO	L1LgS	
MW	W7	WN5	CD3	PSAR	DRACAC	L2LgS	
Surface Tension	W8	WN6	CD4	PHSAR	DRACDO	L3LgS	
Polarizability	D1	IW1	CD5	LgD5	DRDODO	L4LgS	
C ratio	D2	IW2	CD6	LgD6	ACACAC	DD1	
N ratio	D3	IW3	CD7	LgD7	ACACDO	DD2	
NO ratio	D4	IW4	CD8	LgD7.5	ACDODO	DD3	
Num Rings	D5	CW1	HL1	LgD8	DODODO	DD4	
Num Ar Rings	D6	CW2	HL2	LgD9	SOLY	DD5	
	D7	CW3	A	LgD10	LgS3	DD6	
	D8	CW4	CP	AUS7.4	LgS4	DD7	
					LgS5	DD8	

### 3. PCA and PLS Regression Models

QSPR models were built using linear multivariate analysis tools PCA and PLS (Simca *plus Version 10.5*) [28]. All descriptors were transformed with unit variance scaling and mean centering before PCA and PLS analysis. Moreover, the descriptors with a broad range or unequal distribution across the range were logarithmically transformed to obtain better distributions. Three sets of molecular descriptors were assembled for the regression modeling: (1) *ACDlabs* descriptors and *MOE* logS descriptor; (2) *VolSurf+* descriptors; (3) the combination of *ACDlabs*, *MOE* and *VolSurf+* descriptors.

**Figure 2:**
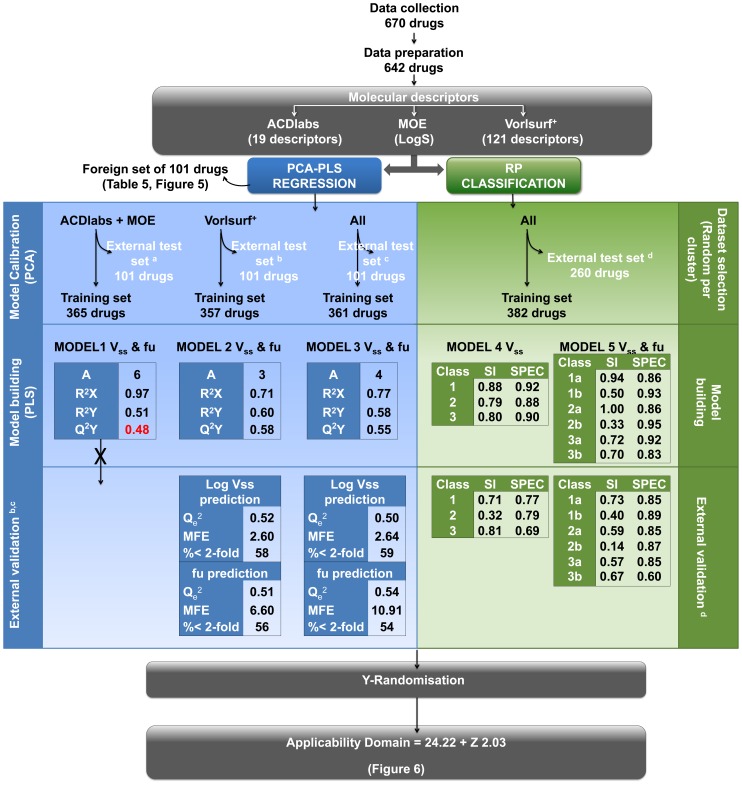
Flowchart of the work process to obtain regression and classification models for V_ss_ and f_u_. MFE =  mean fold error, SI  = Sensitivity, SPEC =  specificity.

**Figure 3:**
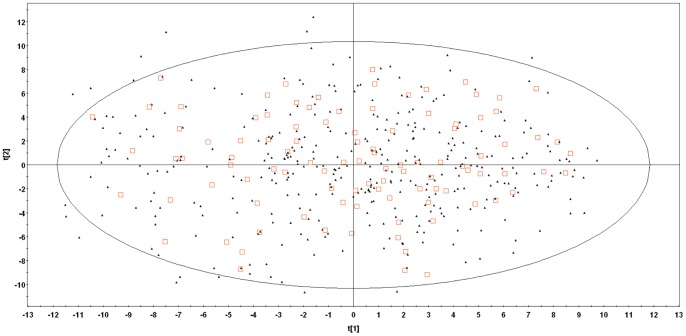
PCA score plot. The final PCA score plot obtained after model 3 calibration where the two principal components explain 27% and 20%, respectively, of the variance in the data set. The open squares represent the drugs in the external test set and the filled triangles the drugs in the training set. The ellipse depicts the 95% confidence region of the model (Hotelling T^2^).

A workflow of the modeling process is presented in Figure 2. Before modeling, a foreign set of 101 drugs was randomly excluded from the final 642 compound set. The descriptor matrix of the remaining 541 drugs was analysed with PCA to identify the drugs that fall outside the general chemical space of the compound set and descriptors that should be excluded from the model (model calibration). Drugs that were outliers based on their distribution in the PCA plot and whose descriptor values fell outside the boundaries outlined in Table 2 were excluded. Based on the scatter plot of the final PCA plot, an external test set (Figure 3) of 101 compounds representative of the chemical space was selected. The external set comprises molecules within the chemical space of the model, while the foreign set, which was selected before the PCA and model calibration, also includes compounds outside the chemical space used for model building. The remaining compounds constitute the training set for the PLS model building (365 drugs for model 1; 357 drugs for model 2; 361 drugs for model 3). The training sets were used to build PLS models that relate the descriptors to the two simultaneously modelled responses, log V_ss_ and f_u_. During initial stages of the analytical process, the number of highly correlated variables observed in the PLS weight plot was gradually reduced in order to equilibrate the influence of the overall set of descriptors on the responses. Subsequent models with improved statistic parameters were obtained and variables deemed least influential to the modelled pharmacokinetic parameters were excluded. The decisions were based on the PLS weight plot and confirmed by the variable importance plot results. Moreover, the distribution of the drugs was followed up by the PLS score and Dmod plots, in order to detect outliers.

**Table 2:** Statistical parameters of the PCA models and the chemical boundaries chosen during the PCA modelling.

	A	R^2^X	Q^2^X	Criteria of model calibration
**Model 1**	7	0.90	0.58	MW^a^<940 PSA^b^<205 POL^c^<71 HBD^d^<10 HBA^e^<15 and -7.71<LogS^f^<0.38
**Model 2**	7	0.79	0.73	MW<940 WO4^g^<100 WO6<2 PSA<205 SOLY^h^<0.93 V^i^<1353 POL<71 LogS9^j^<5.3 W4^k^<483
**Model 3**	7	0.79	0.72	MW<940 WO4<100 PSA<205 SOLY<0.93 MV^l^<466 Rule of 5<3

a **MW:** molecular weight; ^b^ **PSA:** polar surface area; ^c^ **POL:** polarizability; ^d^ **HBD:** hydrogen bond donors; ^e^ **HBA**: hydrogen bond acceptors; ^f^ **LogS:** log of solubility; ^g^ **WO4 and WO6:** hydrogen bond donor volume at different energy levels; ^h^ **SOLY:** intrinsic solubility; ^i^ **V:** molecular volume; ^j^ **LogS9:** log of solubility at pH 9; ^k^ **W4:** hydrophilic volume; **^l^MV:** molar volume.

### 4. Recursive Partitioning Classification Models

A RP analysis was carried out using *Discovery Studio version 3.5* (Accelrys Inc.) to develop decision trees that categorize the compounds into classes that are based on the V_ss_ values or both V_ss_ and f_u_ values (Table 3 and 4). Volume of distribution is defined by drug interactions with the main volumes in the body: extracellular space and cellular tissue space. We used these anatomical volumes as rough guidance to classify the volumes into three classes. Class 1 represents the volume of the extracellular fluid (0–0.3 L/Kg), class 2 represents V_ss_ values that take into consideration distribution to the tissues (0.3–1 L/Kg), and class 3 values of V_ss_ represent significant binding to the cellular components (>1 L/Kg). However, it should be noted that V_ss_ is an apparent volume that does not strictly obey anatomical volumes, therefore the anatomical distribution of the compounds cannot be concluded from the V_ss_. Distribution of compounds into the three classes is shown in Figure 1A. When both V_ss_ and f_u_ values were predicted, each class was further divided into compounds with low to intermediate (<0.7) or high (>0.7) f_u_. Compounds with missing f_u_ values were addressed by assigning them the mean value of all f_u_ values and distributing them equally in the training and external test set, which is a standard approach to handle missing values in RP analysis. In our study, balanced forest of RP was used, since it is the appropriate method for imbalanced data [29]. This type of RP contains a relatively small number of trees (in average 10) using a separate bootstrap sample of the original data for each tree. For each tree, the number of members in all classes is equal to the number of members in the smallest class. The number of descriptors that was used as split criterion within each tree was set to the square root of total descriptors. The weighing method was set to “uniform” and the equalize class sizes to true. All others parameters were set to default.

**Table 3:** Division of training and test set compounds into three classes according to observed V_ss_.

	Class 1	Class 2	Class 3	Total
	V_ss_ = 0–0.3 L/kg	V_ss_ = 0.3–1 L/kg	V_ss_ >1 L/kg	
Training	105	96	181	382
Test	62	71	127	260
Total	167	167	308	642

**Table 4:** Division of training and test set compounds into six classes according to observed V_ss_ and f_u_.

	Class 1a	Class 1b	Class 2a	Class 2b	Class 3a	Class 3b	Tota
	V_ss_ = 0–0.3 L/kg	V_ss_ = 0–0.3 L/kg	V_ss_ = 0.3–1 L/kg	V_ss_ = 0.3–1 L/kg	V_ss_ > = 1 L/kg	V_ss_ > = 1 L/kg	l
	& f_u_ >0.7	& f_u_ <0.7	& f_u_ >0.7	& f_u_ <0.7	& f_u_ >0.7	& f_u_ <0.7	
Training	18	87	22	68	38	149	382
Test	11	51	17	51	21	109	260
Total	29	138	39	119	59	258	642

A training set was used to build the decision trees and an external test set was utilized to evaluate the predictive power of the models. To generate the training and external test set for RP analyses, all compounds were first clustered by similarity based on root mean square deviation and each cluster was divided into training and test sets to ensure that both sets included compounds from each cluster. The data set used to train the model consisted of 382 compounds, while 260 compounds were used as an external test set (Figure 2).

### 5. Model validation

The prediction accuracy of the PLS models was determined by internal and external validation. The internal validation is based on the cross-validation value Q^2^Y (Q^2^) that is calculated by leaving out 1/7 of the data, and predicting these compounds based on a model trained by the remaining data. The external validation is conducted with the external test set. The model was used to predict the log V_ss_ and f_u_ of the external test set. The predicted responses were plotted against the observed responses (i.e. experimental V_ss_ and f_u_). The R^2^ value of the regression line for the plot was considered as the Q_e_^2^ (goodness of prediction of the external test set).

We estimated the predictive ability of the RP classification models using out-of-bag statistics. The external test set was used to estimate the fitting ability of the model on a new dataset that was not used in the model construction. The performance of the RP models is based on three metrics: true positive rate (recall or sensitivity), specificity, and the area under the curve (AUC) of the receiver operating characteristics (ROC) plot [30]. AUC represents the probability that a classifier will be estimated correctly, with values >0.5 indicating better than random prediction and 1 signifying perfect prediction. In the case of more than two classes (multiclassification), a confusion matrix is a square of NxN, where N is the number of classes. AUC is computed as defined by Hand and Till (2001) as an average over components generated from several ROC plots for a Y property and cannot be plotted [30]. For instance, when N (A, B, C) is 3, the classifier's performance is computed per class as follows for class A:
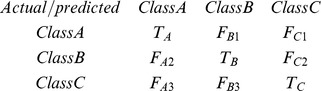


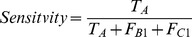


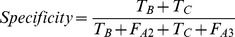



### 6. Y-randomization test

In addition to the internal and external validation, the Y-randomization test (response permutation test) was performed, which estimates the robustness of models [31]. The X data are left intact, whereas the Y data are permuted to appear in a different order (random shuffling). A model is then fitted to the permuted Y-data and the model statistics are computed for the derived model. It is expected that the models from randomized activities would have significantly lower accuracy values.

### 7. The applicability domain of models

An applicability domain (AD) of the model is needed to avoid making predictions for compounds, which differ substantially from the training set molecules. The AD is used to estimate which compounds are suitable for model predictions and avoid unjustified extrapolation of predictions. We used a method introduced by Zhang et al. (2006) for defining the AD based on the distribution of similarities between each compound and its nearest neighbours in the training sets [32]. The AD was calculated as follows:
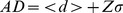



The average of Euclidean distances between all points of the training set were calculated from Similarity and Clustering Canvas of Schrödinger modeling package [33], with 32 bit linear Daylight fingerprint. Data for estimation of the Euclidean distance and application of the AD on new compounds are available in Files S2-S5. Then, using the distances lower than the average, a new average distance <d> and standard deviation σ between these distances were calculated. Z is an arbitrary parameter to control the significance level and considerably affects the number of compounds within the applicability domain. Increasing Z will include compounds that are more dissimilar in the AD. We set the value of Z to 0.7 to calculate the compounds within the AD of the models in the foreign test set.

## Results

### 1. PLS Regression Models

The linear regression model of log V_ss_ and f_u_ was attempted with three descriptor sets: (1) 19 descriptors from *ACDlabs* and *MOE*, (2) 121 descriptors from *VolSurf+* and (3) 140 descriptors from the combination of the two previous sets. The three sets were first analyzed with PCA. In Table 2, the final PCA model statistics for the three strategies are presented as well as the criteria of selection chosen in each case. In Figure 3, the score plot of the final PCA model of data set 3 is shown as an example. Similar plots were obtained for the other data sets.

**Figure 4:**
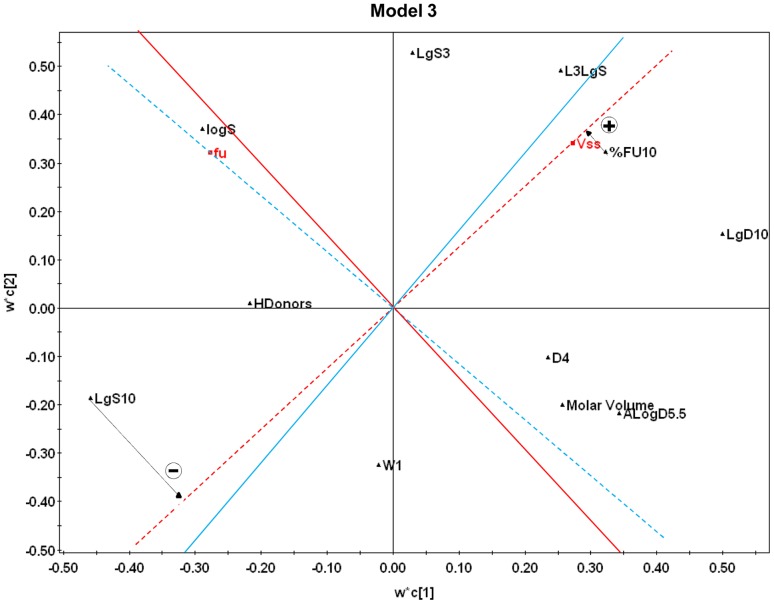
PLS weight plot of model 3. The plot illustrates the relationships between the eleven descriptors (in black) and V_ss_ and f_u_ (in red). The dashed red line crosses the origo and the V_ss_ response, and the continuous red line (perpendicular to the dashed line) represents the borderline between negative and positive influences of the descriptors. The respective lines for f_u_ are blue. Impact of descriptors is interpreted in the following manner: the V_ss_ descriptors that show orthogonal projection on the same side as V_ss_ (on the right from red borderline) have positive impact on V_ss_, and the descriptors on the left side of the borderline show negative impact on V_ss_. The farther away from the origo the projection of the descriptors lies, the stronger is the impact on the corresponding response. As an example of the variablés influence on V_ss,_ two arrows have been drawn that represent the orthogonal projections of variable LgS10 (negatively correlated to V_ss_) and %FU10 (positively correlated to V_ss_). Likewise, the descriptors on the left side of blue borderline show positive impact on f_u_, and the descriptors on the right side of the borderline have negative influence on f_u_.

The statistical values of the final models are present in Figure 2. Model 1 resulted in a non-predictive model, yielding a Q^2^Y smaller than 0.50, and therefore the analysis of this set was not taken any further. The final models were based on 332 compounds and 9 descriptors from *Volsurf+* (model 2) and 353 compounds and 11 descriptors combined from *Volsurf+*, *ACDlabs* and *MOE* (model 3). The PLS weight plot of model 3 is presented in Figure 4, showing the relationships between the X-descriptors and Y-responses, V_ss_ and fu, at the same time. A detailed description of PLS weight interpretation is presented in the legend. The final equations for model 2 and model 3 are:

**Model 2.**

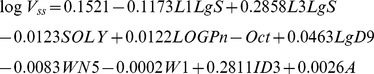


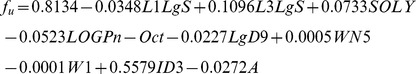



**Model 3.**

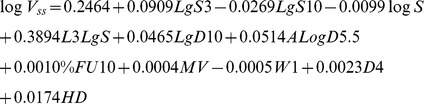


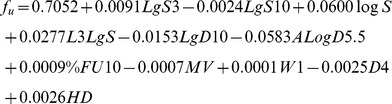
Where L1LgS and L3LgS are solubility profiling coefficients, logS is the logarithm of solubility, LgS3 and LgS10 are the logarithms of solubility at pH 3 and pH 10, respectively, SOLY is intrinsic solubility, LOGP n-Oct is the partitioning coefficient in octanol/water, LgD9, LgD10 and ALogD5.5 are distribution coefficients at pH 9, pH 10 and pH 5.5, respectively, WN5 is hydrogen bond acceptor volume, W1 is hydrophilic volume, ID3 is hydrophobic integy moment, A is amphiphilic moment, %FU10 is % of fraction unionized at pH 10 (not to be confused with f_u_), MV is molar volume, D4 is hydrophobic volume and HD is hydrogen bond donor.

Model 2 and model 3 were internally validated by cross-validation, gaining Q^2^ values of 0.58 and 0.55, respectively. In external validation of the models we determined their accuracy in predicting log V_ss_ and f_u_ with the external test sets. In log V_ss_ prediction by model 2, two outliers were excluded (ribavirin and bilobalide), while in f_u_ prediction by model 2, four outliers (acetylcysteine, amiodarone, aripiprazole, repaglinide) were excluded and in f_u_ prediction by model 3, five outliers were excluded (ethambutol, atovaquone, beclomethasone dipropionate, drotaverine, irbesartan). The statistical results of the predictions are presented in Figure 2. The Y-randomization test after 50 permutations provided R^2^Y- and Q^2^Y-intercepts smaller than the recommended limits of 0.3 and 0.05 for both log V_ss_ and f_u_, respectively (data not shown).

**Figure 5:**
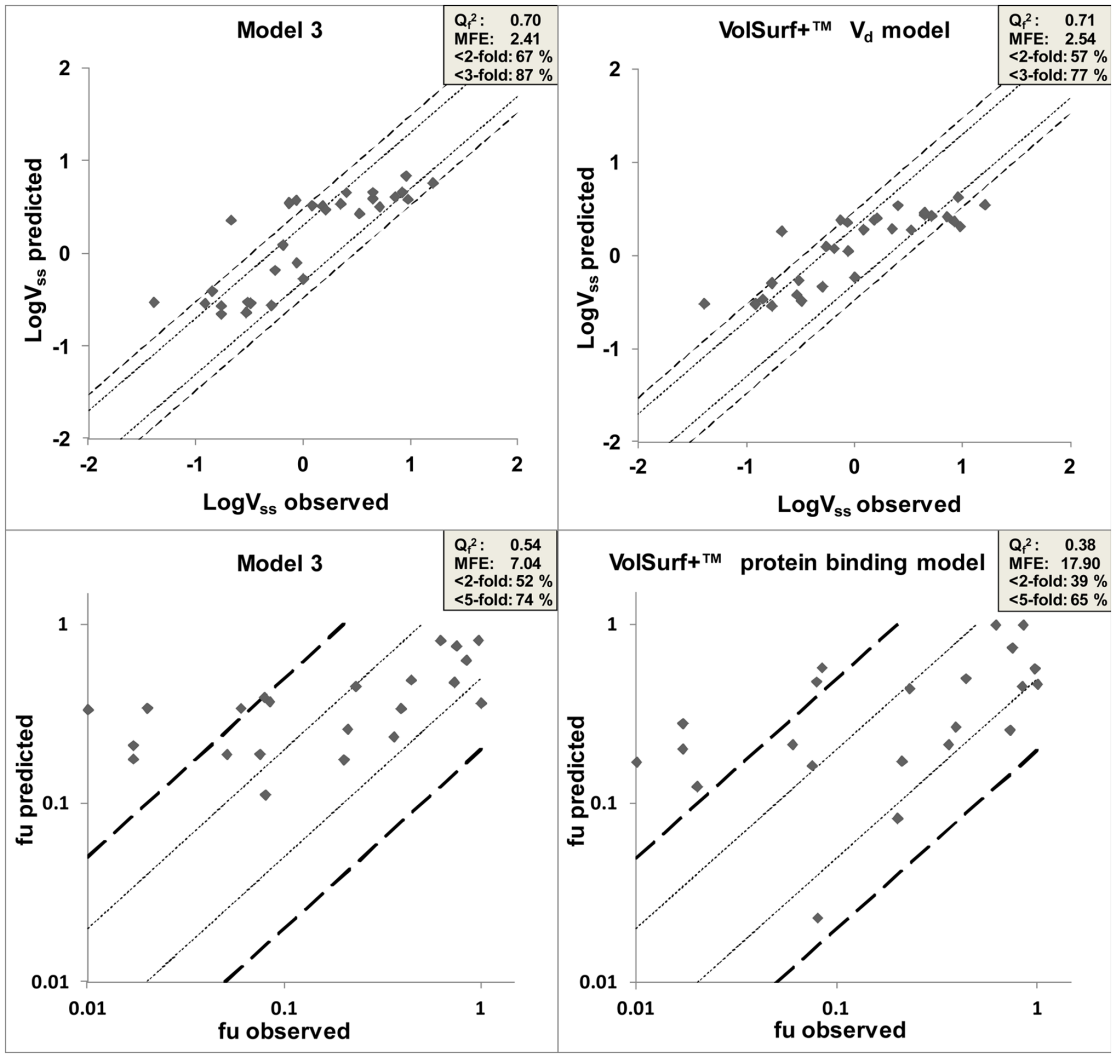
Log V_ss_ and f_u_ prediction plots of model 3 versus *VolSurf+ ADME* models (V_d_ and protein binding). Dot lines represent 2-fold error, dashed lines represent 3 –fold error and long dash lines represent 5-fold error. MFE: mean fold error.

**Figure 6:**
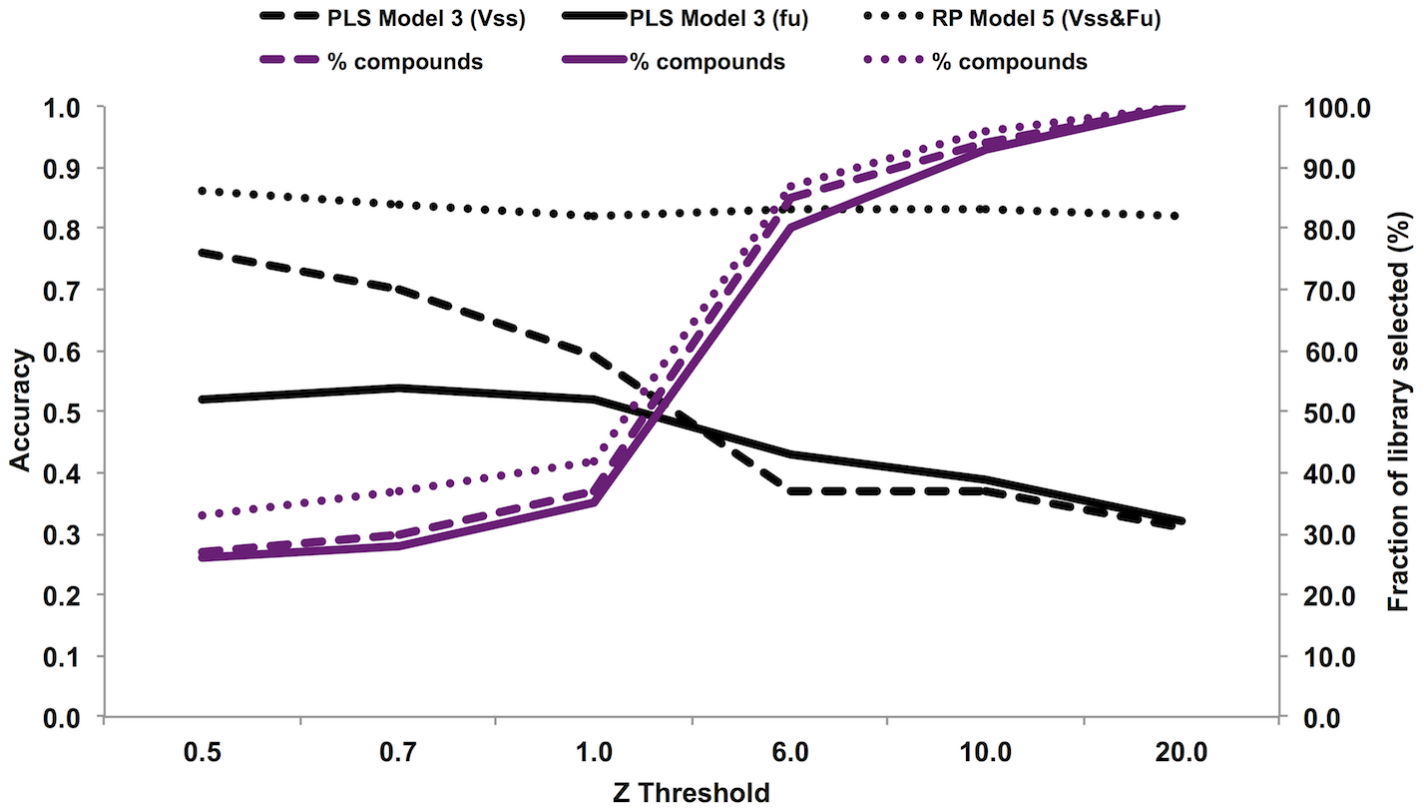
The effect of the AD on the prediction accuracy and chemical space coverage. Dashed black line: Q^2^ of the V_ss_ foreign set predicted with PLS model 3. Dashed purple line: percentage of compounds from the V_ss_ foreign set predicted with PLS model 3. Black line: Q^2^ of the f_u_ foreign set predicted with PLS model 3. Purple line: percentage of compounds from the f_u_ foreign set predicted with PLS model 3. Dotted black line: AUC of the test set predicted with RP classification. Dotted purple line: percentage of compounds from the test set predicted with RP classification.

The AD was estimated from the compounds belonging to the training set as:
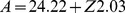



With Z = 0.7, AD is 25.641 that represent the maximum distance between compounds in the training set and new compound to be predicted. The compounds in the foreign test set that fell inside this AD were selected, yielding a set of 35 drugs for model 2, and 30 drugs for model 3. The statistical parameters of log V_ss_ and f_u_ predictions for the foreign set are presented in Table 5 and plots of the observed and predicted responses of model 3 and *VolSurf+* ADME models are presented in Figure 5. A comparison of the predicted and the observed values is found Table S1. Increasing Z increases the number of compounds in the foreign test set that are considered to be within the applicability domain but decreases the accuracy of prediction due to inclusion of dissimilar nearest neighbors (Figure 6).

**Table 5:** Statistical parameters for log Vss and f_u_ predictions of the foreign set compounds inside the applicability domain of the models, calculated with Z = 0.7.

	Log V_ss_ prediction of foreign set			f_u_ prediction of foreign set		
	Q_f_^2^	MFE	% <2-fold	Q_f_^2^	MFE	%<2-fold
**Model 2**	0.62	2.85	60	0.59	5.58	54
**Model 3**	0.70	2.41	67	0.54	7.04	52

### 2. RP Classification Models

The AUC for the in-bag training data for all trees in the forest model is 0.96 and 0.92, and the out-of-bag AUC is 0.81 and 0.79 for the V_ss_ and V_ss_ & f_u_ models, respectively. The in-bag results use predictions for the records used to train the tree, while the out-of-bag results use predictions for the left-out records. The statistics for the training set data presented in Figure 2 are derived from the in-bag results. The external test set including 260 compounds (described in Methods section) was used to evaluate the predictive ability of the two models. All compounds were classified according to their V_ss_ or V_ss_ & f_u_ values without applying AD. The overall prediction accuracy, calculated as ROC curve, was 0.78 and 0.82, respectively, and the sensitivity and specificity values are presented in Figure 2. The confusion matrices are presented in Tables S2-S7.

In general, the sensitivity of the models is high for compounds with a very low or high volume of distribution, while compounds belonging to class 2, with V_ss_ values between 0.3 and 1 L/kg are more difficult to classify correctly. The V_ss_ model performed well on the training set, with sensitivity 0.79 in class 2, but less than half of the class 2 compounds in the training set (42 of 93 compounds, leading to a sensitivity of 0.45) were predicted to the correct class in the out-of bag results (Table S3). Similarly, the model was able to identify class 1 and class 3 compounds form the external test set (sensitivity 0.71 and 0.81, respectively), while recognition of class 2 test set compounds was not as successful (sensitivity 0.32, Figure 2, Table S6). Interestingly, in the V_ss_ & f_u_ model, compounds with high f_u_ were predicted more accurately, with 10 of 17 compounds of the test set compounds correctly classified (sensitivity 0.59), but only 7 of 51 compounds with low f_u_ (sensitivity 0.14) (Figure 2, Table S7). The Y-randomization test was performed four times, and the AUC values for the model using the data set with experimental V_ss_ and V_ss_ & f_u_ values were significantly higher than those obtained from the dataset with randomized values (data not shown), indicating that our models are statistically robust. The AD was applied to the test set and its effect analyzed on the V_ss_ & f_u_ model (Figure 6). The prediction accuracy was highest with low Z cutoff, as expected, and slowly decreasing as the cutoff was increased to 1. However, increasing the cutoff from 1 to 20 did not markedly affect the prediction accuracy, while increasing the coverage of the test set from 39% to 100%. The small decrease in prediction accuracy is probably due to the cluster-based approach used to select the training and test set (described in Methods) that make the chemical space covered by two set similar.

One aid for interpretation of forest models is a set of descriptor importance measures, which indicate the relative importance of the descriptors in distinguishing among the different classes in the data. The percent selection frequency empirically appears to best distinguish truly important descriptors from others. It represents the percent of the time that the descriptor was selected for a split when a split was possible. A summary of descriptors ranked as top 10 based on their frequency of occurrences in the models are given in Table 6. It should be noted that size, polarity and lipophilicity are predominant in all models. The simple importance measures reported here are known to have bias in some cases [34]. However, if all descriptors have the same character as in our cases (e.g. they are all continuous numerical properties), then bias is generally not an issue.

**Table 6:** Most influential descriptors in the classification models.

V_ss_				V_ss_ and f_u_			
Descriptor	Type	Number of Chances	Percent Selection Frequency	Descriptor	Type	Number of Chances	Percent Selection Frequency
Rule Of 5	Drug like	12	8.3	PSAR	Polar area	4	25
CD3	Hydrophobic area	31	6.4	Num Rings	Topology	5	20
FLEX_RB	Size/Shape	69	5.8	W8	Hydrophilic area	5	20
L1LgS	Solubility	53	5.7	R	Size/Shape	10	10
CD6	Hydrophobic area	37	5.4	DRDRDO	Pharmacoforic	12	8.3
CW8	Hydrophilic area	20	5	D8	Hydrophobic area	26	7.7
LgS11	Solubility	66	4.5	LgD9	LogD	32	6.3
C ratio	Topology	45	4.4	ALogD5	LogD	34	5.9
NO ratio	Topology	48	4.2	CP	Shape	17	5.9
LgS5	Solubility	72	4.2	AUS7.4	Charge	35	5.7

## Discussion

We have predicted V_ss_ and f_u_ with linear PLS models and nonlinear RP classification models, aiming for models that rely on *in silico* descriptors only and therefore are suitable for screening. V_ss_ is affected by the f_u_ in plasma, and we wanted to explore if predicting both parameters in parallel would help to find relevant physicochemical descriptors affecting these parameters. PLS can easily be used to correlate descriptors with several related responses, but to our knowledge, this approach has not been used in pharmacokinetic QSPR modeling.

The RP classification model was reasonably successful in classifying compounds with high (≥1 L/kg) or low (0–0.3 L/kg) V_ss_, while it had difficulties to identify the compounds with moderate (0.3–1 L/kg) V_ss_. Interestingly, the level of binding to plasma proteins had an influence on the prediction accuracy, which was seen most clearly in the moderate V_ss_ class, where compounds with high f_u_ were correctly predicted in 59% of the test set, but only 14% of those with low f_u_ (Figure 2, Table S7). The attempt to create a PLS model for V_ss_ and f_u_ (model 1) starting with only 19 descriptors from *ACDlabs* and *MOE* was not successful, but using a wider range of descriptors from *Volsurf+* resulted in a predictive model (model 2) (Table 1 and Figure 2). The combination of all descriptors to model 3 did not significantly improve the prediction of the external set (V_ss_ Q_e_^2^ = 0.50, f_u_ Q_e_^2^ = 0.54) compared to model 2 (V_ss_ Q_e_^2^ = 0.52, f_u_ Q_e_^2^ = 0.51) (Figure 2). However, model 3 had better success in predicting the V_ss_ of the compounds in the foreign set (model 3 Q_f_^2^ = 0.70, model 2 Q_f_^2^ = 0.62) (Table 5). Notably, the prediction of the compounds in the foreign set within the AD was better than for the external set for both model 2 and 3. The AD was not used to filter compounds for prediction in the external set, which might be one reason for the improved performance on the foreign set. The use of an AD prevents extrapolation beyond the limits of chemical space that was used to build the model and can be used to identify the compounds for which predictions are reliable.

The impact of the descriptors on the responses can be observed graphically in the PLS weight plot (model 3 in Figure 4, model 2 in Figure S1). In model 3, the descriptors L3LgS, %FU10 and LgD10 have the highest positively correlated impact to V_ss_ (L3LgS, LogP n-oct and LgD9 in model 2, Figure S1), while LgS3, D4, Molar Volume and ALogD5.5 have a more moderate positive influence on V_ss_ (A in model 2). LgS10 has the largest negative correlation to V_ss_ (L1LgS in model 2), while W1, HDonors and LogS have smaller negative correlation in model 3 (Wn5, SOLY, W1 and ID3 in model2). On the other hand, LogS, LgS3 and LgS10 have the highest positive correlation with f_u_ (SOLY in model 2), while LogD10 and AlogD5.5 have the highest negative correlation (LogD9 and LOGP n-Oct in model 2). All in all, this suggests that charge and lipophilicity of the drug affect drug distribution, albeit with an inverse relationship. Thus, the lipophilicity descriptors have high correlation with the two responses, positive with V_ss_ and negative with f_u_, while reversely, the charge and solubility descriptors have negative correlation with V_ss_ and positive with f_u_. There is a complex relationship between f_u_ and V_ss_ and increasing the f_u_ of a compound does not inevitably lead to a higher volume of drug distribution, as is stated in many pharmacokinetic textbooks [35], [36]. This is easy to understand, since structural changes influencing the drugs ability to bind to plasma proteins may also affect the tissue binding of the drug.

Similar descriptors were found to be important in both the RP classification models (Table 6) and the PLS models. These include solubility descriptors, LogD at pH 9 or 5, as well as hydrophilic and hydrophobic area and volume descriptors. Due to the complexity of V_ss_ and f_u_, many descriptors were always required to yield good prediction capability. Previously, trends have been observed between V_ss_ and LogP, polar surface area and hydrogen bond descriptors for the data set we have used [18]. Using the same data set, Berellini et al. (2009) found hydrogen bonding, LogD at pH 5–10, flexibility of the molecule and the *Volsurf+* descriptors DRDRDO, DRDRAC to be important in their V_ss_ model [12]. DRDRDO and DRDRAC are pharmacophoric descriptors of the maximum area of the triangles derived from Dry (DR), H-bond acceptor (AC) and H-bond donor (DO) points in a molecule. DRDRDO and flexibility were among the ten most influential descriptors in the RP models, but in the PLS models they did not have equally high importance. However, when comparing our descriptor selection to previous models of V_ss_ it must be kept in mind that we have modeled both V_ss_ and f_u_ parameters. Therefore a comparison is not directly applicable as descriptors having high influence on one parameter, but no correlation with the other parameter, are likely to be removed in our models.

Outliers are usually interesting, and the analysis of outliers can sometimes give a deeper understanding of the mechanisms under investigation. However, it is difficult to analyse the outliers in this study, because we do not know the reason for their exceptional behavior. Deviations in V_ss_ may be due to the active transport (influx or efflux) or compound specific binding to the tissues. As an example, let's consider the outliers in the prediction of V_ss_ by the PLS models (ribavirin, bilobalide, tamsulosin, decitabine). Ribavirin and decitabine are substrates of widely expressed nucleoside transporters, and extensive active transport might lead to outlier profiles of ribavirin and decitabine [37]. Tamsulosin is a substrate of alpha1 adrenergic receptors and bilobalide binds to GABA, glycine, and 5-HT3 receptors [38]. We cannot be sure, however, if these transport and binding phenomena take place substantially enough to cause exceptional V_ss_ values. Clearly, V_ss_ and f_u_ are complex phenomena that are affected by numerous factors. Therefore, explanations for the outlier behavior are not on firm ground and the reasons can be identified only by extensive experimental work.

We compared the performance of our model 3 with the volume of distribution and plasma protein binding models available in the *Volsurf+* package (Figure 5). As no AD is reported for the *Volsurf+* models, we have applied our AD with the Z cutoff value of 0.7 to select the compounds from the foreign test set for both models. For the practical use of AD in V_ss_ and f_u_ prediction, see File S2. It should be noted that we are not aware of which compounds have been used to train the *Volsurf+* model, and it is possible that some, or all, of the compounds used in our test set have been used to for that purpose. The same considerations apply for the *Volsurf+* plasma protein binding model. Our model achieved higher accuracy than the *Volsurf+* model in predicting f_u_ (Q_f_^2^ = 0.54 and Q_f_^2^ = 0.38, respectively) (Figure 5), while the prediction of V_ss_ was comparable to the *Volsurf+* model (V_ss_ Q_f_^2^ = 0.70 and Q_f_^2^ = 0.71 for model 3 and *Volsurf+* models, respectively). The best predictions with our model were obtained at f_u_ values above 0.05. Predictions of the compounds with f_u_ values above 0.05 in the foreign set had a MFE of only 2.2 for model 3, compared to 7.04 for the whole foreign set (Figure 5, table 5). The predictions at f_u_ values below 0.05 give high FE values (>5-fold), whereas % error in this region is low. However, FE is pharmacologically a more relevant parameter, because the free drug concentration in plasma, C_u_, is defined as f_u_ x C. Therefore, 3-fold change in f_u_ is expected to result in 3 fold change in C_u_ Unfortunately, we do not have an explanation for the poor results for the compounds that have very low f_u_ values, however, the compounds that were badly predicted by our models were also badly predicted by the *Volsurf+* model (Table S1), suggesting that the exceptional behavior is drug dependent and not due to the model.

The physical complexity of the V_ss_ and f_u_ parameters makes their prediction very challenging, and we were not able to reach models with optimal predictability. One way to improve prediction accuracy is to build the model using a narrower range of more similar compounds. We divided the data set of 642 compounds based on structural features or chemical properties and used these data sets to build several sub-models. However, the models were not able to achieve much higher accuracy than the more global models presented here (data not shown), but presented a much narrower AD and therefore more limited use.

## Conclusions

The PLS models of V_ss_ showed similar performance to the commercial *Volsurf+* model, while the f_u_ prediction accuracy was slightly better. The RP classification models were able to distinguish between compounds with high or low V_ss_ values, but accurate classification of moderate V_ss_ or of low f_u_ values were not as successful. Due to the complex nature of V_ss_ and f_u_ parameters, a fairly large number of descriptors were needed for meaningful models. The advantages of the models compared to previous models is that they are based on a large set of structurally unrelated compounds, they are open, and they have a defined AD, which aids in identifying compounds for which reliable predictions can be made.

## Supporting Information

Figure S1 **PLS model 2 weight plot.**
(TIF)

Table S1 **Table of predicted vs. observed values for foreign set with PLS models.**
(XLSX)

Table S2 **Confusion matrix in-bag training results for the V_ss_ classification model.**
(DOCX)

Table S3 **Confusion matrix out-of-bag training results for the V_ss_ classification model.**
(DOCX)

Table S4 **Confusion matrix in-bag training results results for the V_ss_ & f_u_ classification model.**
(DOCX)

Table S5 **Confusion matrix out-of-bag training results results for the V_ss_ & f_u_ classification model.**
(DOCX)

Table S6 **Confusion matrix external test results for the V_ss_ classification model.**
(DOCX)

Table S7 **Confusion matrix external test results for the V_ss_ and f_u_ classification model.**
(DOCX)

File S1 **Final data set used for models.**
(SDF)

File S2 **Instructions for use of applicability domain.**
(DOCX)

File S3 **Training set for RP models.**
(SDF)

File S4 **Training set for PLS model 2.**
(SDF)

File S5 **Training set for PLS model 3.**
(SDF)
