# Correction: Applying Linear and Non-Linear Methods for Parallel Prediction of Volume of Distribution and Fraction of Unbound Drug

**DOI:** 10.1371/journal.pone.0141943

**Published:** 2015-10-28

**Authors:** Eva M. del Amo, Leo Ghemtio, Henri Xhaard, Marjo Yliperttula, Arto Urtti, Heidi Kidron

S4 File in the Supporting Information is an incorrect file. Please view the correct S4 File here.

## Supporting Information

S4 File Training set for PLS model 2. (SDF)
