# Supplementary figures and images for: Applying Linear and Non-Linear Methods for Parallel Prediction of Volume of Distribution and Fraction of Unbound Drug
Source: PLoS One. 2013 Oct 7;8(10):e74758. doi: 10.1371/journal.pone.0074758 (PMC3792104; doi:10.1371/journal.pone.0074758)

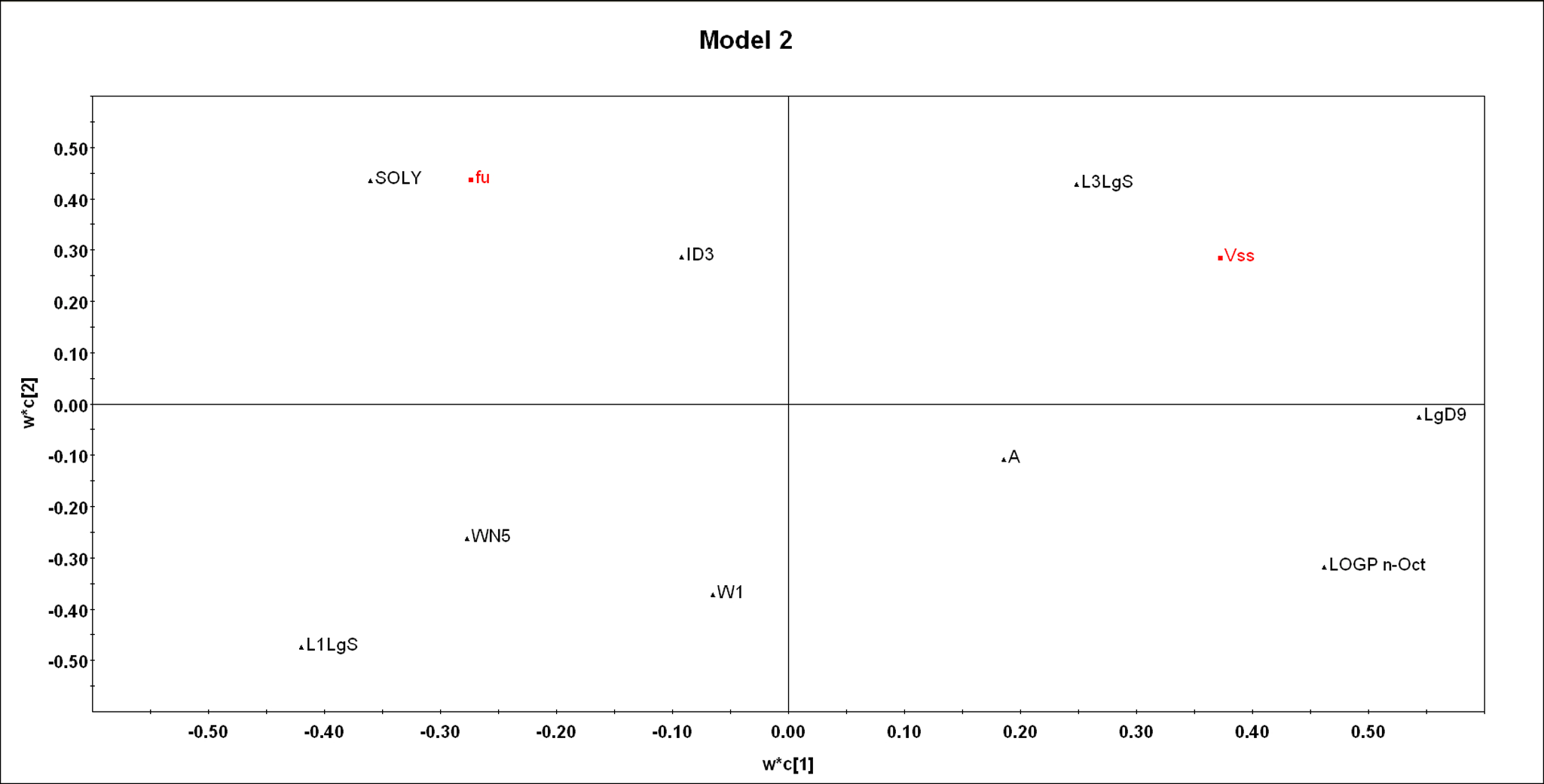

Supplement: Figure S1 — PLS model 2 weight plot. (TIF) [file pone.0074758.s001.tif]
