# Supplementary material for: Applying Linear and Non-Linear Methods for Parallel Prediction of Volume of Distribution and Fraction of Unbound Drug
Source: PLoS One. 2013 Oct 7;8(10):e74758. doi: 10.1371/journal.pone.0074758 (PMC3792104; doi:10.1371/journal.pone.0074758)
Supplement: Table S2 — Confusion matrix in-bag training results for the Vss classification model. (DOCX) [file pone.0074758.s003.docx]

**Table S2:** Confusion matrix training results for the V_ss_ classification model (In-bag training data results for 10 trees)

| Actual\Predicted  class | 1 | 2 | 3 |
| --- | --- | --- | --- |
| 1 | 89 | 7 | 5 |
| 2 | 8 | 74 | 12 |
| 3 | 12 | 25 | 150 |
