# Supplementary material for: Applying Linear and Non-Linear Methods for Parallel Prediction of Volume of Distribution and Fraction of Unbound Drug
Source: PLoS One. 2013 Oct 7;8(10):e74758. doi: 10.1371/journal.pone.0074758 (PMC3792104; doi:10.1371/journal.pone.0074758)
Supplement: Table S3 — Confusion matrix out-of-bag training results for the Vss classification model. (DOCX) [file pone.0074758.s004.docx]

**Table S3:** Confusion matrix training results for the V_ss_ classification model (Out-of-bag training data results for 10 trees)

| Actual\Predicted  class | 1 | 2 | 3 |
| --- | --- | --- | --- |
| 1 | 76 | 17 | 8 |
| 2 | 20 | 42 | 31 |
| 3 | 23 | 35 | 129 |
