# Supplementary material for: Applying Linear and Non-Linear Methods for Parallel Prediction of Volume of Distribution and Fraction of Unbound Drug
Source: PLoS One. 2013 Oct 7;8(10):e74758. doi: 10.1371/journal.pone.0074758 (PMC3792104; doi:10.1371/journal.pone.0074758)
Supplement: Table S4 — Confusion matrix in-bag training results results for the Vss & fu classification model. (DOCX) [file pone.0074758.s005.docx]

**Table S4:** Confusion matrix training results results for the V_ss_ & f_u_ classification model (In-bag training data results for 10 trees)

| Actual\Predicted  class | 1 | 2 | 3 | 4 | 5 | 6 |
| --- | --- | --- | --- | --- | --- | --- |
| 1 | 17 | 1 | 0 | 0 | 0 | 0 |
| 2 | 20 | 38 | 6 | 2 | 1 | 9 |
| 3 | 0 | 0 | 22 | 0 | 0 | 0 |
| 4 | 6 | 6 | 9 | 21 | 9 | 12 |
| 5 | 0 | 0 | 6 | 0 | 27 | 4 |
| 6 | 2 | 5 | 4 | 7 | 14 | 75 |
