# Supplementary material for: Applying Linear and Non-Linear Methods for Parallel Prediction of Volume of Distribution and Fraction of Unbound Drug
Source: PLoS One. 2013 Oct 7;8(10):e74758. doi: 10.1371/journal.pone.0074758 (PMC3792104; doi:10.1371/journal.pone.0074758)
Supplement: Table S5 — Confusion matrix out-of-bag training results results for the Vss & fu classification model. (DOCX) [file pone.0074758.s006.docx]

**Table S5:** Confusion matrix training results for the V_ss_ & f_u_ classification model (Out-of-bag training data results for 10 trees).

| Actual\Predicted  class | 1 | 2 | 3 | 4 | 5 | 6 |
| --- | --- | --- | --- | --- | --- | --- |
| 1 | 11 | 3 | 1 | 1 | 2 | 0 |
| 2 | 31 | 26 | 4 | 7 | 4 | 15 |
| 3 | 3 | 1 | 12 | 3 | 2 | 1 |
| 4 | 8 | 6 | 17 | 12 | 2 | 23 |
| 5 | 2 | 4 | 12 | 1 | 12 | 7 |
| 6 | 4 | 7 | 6 | 14 | 18 | 100 |
