# Supplementary material for: Applying Linear and Non-Linear Methods for Parallel Prediction of Volume of Distribution and Fraction of Unbound Drug
Source: PLoS One. 2013 Oct 7;8(10):e74758. doi: 10.1371/journal.pone.0074758 (PMC3792104; doi:10.1371/journal.pone.0074758)
Supplement: Table S6 — Confusion matrix external test results for the Vss classification model. (DOCX) [file pone.0074758.s007.docx]

**Table S6:** Confusion matrix external test results for the V_ss_ classification model.

| Actual\Predicted  class | 1 | 2 | 3 |
| --- | --- | --- | --- |
| 1 | 41 | 15 | 2 |
| 2 | 22 | 23 | 27 |
| 3 | 13 | 21 | 96 |
