# Supplementary material for: Applying Linear and Non-Linear Methods for Parallel Prediction of Volume of Distribution and Fraction of Unbound Drug
Source: PLoS One. 2013 Oct 7;8(10):e74758. doi: 10.1371/journal.pone.0074758 (PMC3792104; doi:10.1371/journal.pone.0074758)
Supplement: Table S7 — Confusion matrix external test results for the Vss and fu classification model. (DOCX) [file pone.0074758.s008.docx]

**Table S7:** Confusion matrix external test results for the V_ss_ and f_u_ classification model.

| Actual\Predicted class | 1 | 2 | 3 | 4 | 5 | 6 |
| --- | --- | --- | --- | --- | --- | --- |
| 1 | 8 | 1 | 2 | 0 | 0 | 0 |
| 2 | 13 | 20 | 1 | 2 | 3 | 12 |
| 3 | 4 | 0 | 10 | 0 | 2 | 1 |
| 4 | 5 | 6 | 4 | 7 | 4 | 25 |
| 5 | 0 | 2 | 6 | 0 | 12 | 1 |
| 6 | 0 | 5 | 4 | 15 | 12 | 73 |
