# Supplementary material for: Applying Linear and Non-Linear Methods for Parallel Prediction of Volume of Distribution and Fraction of Unbound Drug
Source: PLoS One. 2013 Oct 7;8(10):e74758. doi: 10.1371/journal.pone.0074758 (PMC3792104; doi:10.1371/journal.pone.0074758)
Supplement: File S2 — Instructions for use of applicability domain. (DOCX) [file pone.0074758.s010.docx]

File S2. **Instructions to estimate whether new compounds belong to the AD**

For new compounds, average Euclidean distance between each compound and all compounds of the training set is first calculated from Similarity and Clustering Canvas of Schrödinger modeling package, with 32 bit linear Daylight fingerprint. The training sets are found in Supplementary material S4 and S5. Then, using average Euclidean distances, predictions for compounds with distances equal or lower than the calculated AD are considered reliable, while predictions for compounds with distances higher than the AD should be considered more unreliable.
